# AN ESSENTIAL TOOL TO BE OPTIMIZED: SYNDROMIC MANAGEMENT OF VAGINAL DISCHARGE IN HAITI

**DOI:** 10.21010/Ajidv18i1.5

**Published:** 2023-10-20

**Authors:** --STURGIS, Morgan, -- BUCKMAN, Victoria, --DIAWARA, Samiya, FIQUITA St. Paul, F, ADRIEN Marlene, M, ZHAO, Xixi, MEHTA, Supriya, TOWBIN, Jennifer, CRANE, Stephanie

**Affiliations:** * Rush Medical College, Chicago IL USA; † Stanford Health Care, Palo Alto CA USA; †† University of Illinois School of Public Health, Chicago IL USA; # Rush Department of Internal Medicine, Chicago IL USA

**Keywords:** Haiti, syndromic management, vaginal discharge syndrome, community health

## Abstract

**Background:**

Haiti, like many low-income countries in crisis, has limited resources for etiologic diagnosis of vaginal discharge. As such, we sought to characterize variability in diagnoses of women presenting with vaginal discharge syndrome, with the goal to improve standardization of syndromic management.

**Materials and Methods:**

Participants aged 18 years and older endorsing vaginitis, or dysuria were recruited at Jerusalem Clinic over two, one-week periods in April 2018 and July 2019. We calculated Spearman rank correlations among history, exam findings, and diagnoses based on clinical presentation, to understand presentation groupings and their management.

**Results:**

Among 98 women, median age was 33.5 years, and most frequent symptoms were: vaginal discharge (97%), vaginal itch (73%), and/or suprapubic pain (68%). Most common physical exam findings were vaginal discharge (86%), suprapubic/lower quadrant tenderness (29%), cervical motion tenderness (24%), and cervical erythema (20%). Most symptoms and physical exam findings were weakly correlated with each other. Nearly one-third (31%) were diagnosed with normal physiologic vaginal discharge or no diagnosis, followed by Bacterial vaginosis (31%), vulvovaginal candidiasis (15%), cervicitis/PID (13%), and STI (7%). No reported symptoms strongly differentiated diagnostic categories. Diagnoses varied considerably by exam findings.

**Conclusions:**

The weak correlations between symptoms, exam findings, and diagnoses could represent variability in assessment. In the absence of reliable and accessible laboratory testing, the importance of standardizing syndromic management becomes increasingly relevant. Results from our study support the utility of speculum examination and more standardized documentation of physical exam findings. Next steps include the development of local algorithms to promote standardization of treatment of vaginal discharge syndrome.

## Introduction

Sexually transmitted infections (STIs) are a major public health burden, with over 350 million new cases each year of curable STIs, including *C. trachomatis* (CT)*, N. gonorrhoeae* (NG), and *T. vaginalis* (TV), all of which can present as vaginal discharge syndrome in affected women (“Web Annex”, 2021). Vaginal discharge is also common among women with Bacterial vaginosis (BV), the most common vaginal condition among reproductive age women, with general population prevalence ranging from 23-29% (Peebles, 2019). While all these conditions can have overlapping presentations, they each have distinct antimicrobial treatment regimens, highlighting the importance of accurate diagnosis for effective cure (Workowski, 2021; Unemo, 2020). However, in resource constrained settings, there is limited availability for etiologic testing, and treatment often relies on syndromic diagnosis. Syndromic management relies on signs and symptoms to guide treatment decisions (“Guidelines”, 2021). Unfortunately, it does not perform well compared to etiologic testing. In systematic review and meta-analysis, vaginal discharge syndromic management had poor diagnostic performance with sensitivity ranging 27-90% and specificity ranging 35-85% for cervical infections (CT and NG), thus many women are either over- or under- treated using syndromic management (Zemouri, 2016). However, in the absence of reliable etiologic testing, the adaptation of local syndromic management flow charts may improve diagnostic performance, over World Health Organization (WHO) flow charts (van Gemert, 2018). Haiti, like many other low- and middle- income countries (LMIC), has limited resources for etiologic diagnosis regarding vaginal discharge. One study conducted in 2013 in rural Haiti incorporated laboratory diagnosis and found that among 206 women presenting with gynecological symptoms, BV was the most common condition (28.3%) followed by CT (5.4%), NG (1.0%), and candidiasis (2.2%), with no cases of TV (Bristow, 2014). There is very limited data available in Haiti to further inform local epidemiology or syndromic management algorithms. Haiti remains the poorest country in the western hemisphere, ranking 20^th^ in maternal mortality and with 14% of mothers having early childbirth before age 18 (“Country Profiles: Haiti”, 2023). As of January 2023, Haiti officially has no functioning parliament, no constitutional representation at any state level and no clear path forward. Rival gangs control much of Port-au-Prince, ruling by terror and intimidation. In the wake of what many believe to be a failed state, opportunity to build infrastructure and laboratory capacity while critically needed is unlikely to be in place soon. As such, the importance of optimizing and standardizing syndromic management becomes increasingly relevant. We sought to characterize variability in diagnoses of women presenting with vaginal discharge syndrome, with the eventual goal to improve standardization of syndromic management, and improved antibiotic stewardship.

## Materials and Methods

This study was approved by the Institutional Review Board at Rush University Medical Center. The study was conducted in Jerusalem, Haiti, a post- 2010 earthquake resettled population of ~300,000, located 18 km from Port-au-Prince (“Community Empowerment”, 2022). Participants were recruited at Jerusalem Clinic, a standalone clinic, over two, one-week periods in April 2018 and July 2019, utilizing a convenience sample with continuous enrollment. Eligible women were aged 18 years and older who presented to the clinic with complaints of vaginitis or dysuria. A native Creole speaker employed as a health worker translated for clinic physicians to assess eligibility and obtain informed consent. Consented participants completed an intake survey administered and collected by the native Creole speaker to assess demographic information, symptoms, and sexual and hygiene practices before being seen by a provider who completed a pelvic exam with speculum. The goal of this analysis was to identify patient factors (history, exam findings) that were most variable in terms of clinician diagnosis in the absence of laboratory testing. The goal is to provide feedback and develop a local algorithm to help standardize the assessment and treatment of vaginal discharge syndrome in the absence of laboratory testing. To identify sources of variability, we compared frequencies of diagnoses by history and exam findings. When categorizing provider diagnosis for analysis, if a condition was suspected (e.g., “normal physiologic discharge vs. vaginitis”), the suspected pathologic diagnosis was recorded (i.e., BV/vaginitis). Where “unspecified” or “vaginal discharge” (not further specified) was diagnosed, this was categorized for analysis as “no diagnosis”. In instances where a specific STI was indicated (e.g., “trichomoniasis”, “gonorrhea/chlamydia”, “HSV”), this was categorized as “STI” for analysis. Pelvic inflammatory disease (PID; n=2) was analyzed together with cervicitis (n=12) due to sparsity. For two women, more than one potential diagnosis was listed, and both diagnoses were categorized. Next, we estimated Spearman rank correlations among history, exam findings, and provider diagnoses, to understand presentation groupings and how they were being managed by providers.

## Results

A total of 98 women consented to participate in our study with a median age of 33.5 years (interquartile range 27 – 42 years; Table 1). Symptom duration was collected as a free text field and categorized as days (6%), weeks (10%), months (37%), or years (46%), with over half (57%) of participants having previously seen a provider for their symptoms. The most frequent symptoms reported were: vaginal discharge (97%), vaginal itch (73%), vaginal burning/pain (63%), burning with urination (60%), and/or suprapubic pain (68%) (Table 1). The most common physical exam findings were vaginal discharge (86%), suprapubic/lower quadrant tenderness (29%), cervical motion tenderness (24%), cervical erythema (20%), and odor on exam (12%).

**Table 1:** Distribution of demographic, behavioral and symptom presentation from April 2018 and July 2019.

	N=98 n (%)
Age in years, median (interquartile range [IQR])	33.5 (27 – 42)
Age	
18-25	21 (21.4)
26-35	30 (30.6)
36-45	30 (30.6)
46-77	17 (17.4)
Age in years started having sex, median (IQR)	18 (17-20)
Lifetime number of sex partners	
0	2 (2.0)
1	39 (39.8)
2	25 (25.5)
3	21 (21.4)
4 or more	11 (11.2)
Condom use (n=2 missing)	
No	61 (63.5)
Yes	35 (36.5)
Duration of symptoms (n=1 missing)	
Days	6 (6.2)
Weeks	10 (10.3)
Monthsāā	36 (37.1)
Years	45 (46.4)
Symptoms, present	
Vaginal discharge	95 (96.9)
Foul odor (n=1 missing)ā	54 (55.7)
Vaginal itching	72 (73.5)
Vaginal dryness	49 (50.0)
Vaginal spotting of blood	21 (21.4)
Vaginal burning or pain	62 (63.3)
Burning with urination	59 (60.2)
Pain with sex (n=6 missing)	47 (51.1)
Suprapubic pain	67 (68.4)
Nausea and/or vomiting (n=2 missing)	37 (38.5)
Have you seen a doctor for this before	
No	42 (42.9)
Yes	56 (57.1)
Physical Exam Findings	
Vaginal discharge (n=2 missing)	84 (85.7)
Odor on exam (n=2 missing)	12 (12.2)
Cervical erythema (n=5 missing)	20 (20.4)
Bleeding from cervix (n=5 missing)	11 (11.2)
Cervical motion tenderness (n=5 missing)	24 (24.5)
Vesicles or ulcers (n=3 missing)	3 (3.1)
Suprapubic/lower quadrant pain (n=5 missing)	27 (28.6)
Diagnosis	
Normal/physiologic vaginal discharge, vaginal discharge unspecified, “NA” (Not applicable)	30 (30.6)
Bacterial vaginosis	30 (30.6)
Vulvovaginal candidiasis	15 (15.3)
Cervicitis or Pelvic Inflammatory Disease	13 (13.3)
Sexually transmitted infection	7 (7.1)

This table represents demographic, behavioral and symptom presentations in the left column, and percentiles in the right column.

### Correlation of Reported Symptoms with Physical Exam Findings

**Figure 1:**
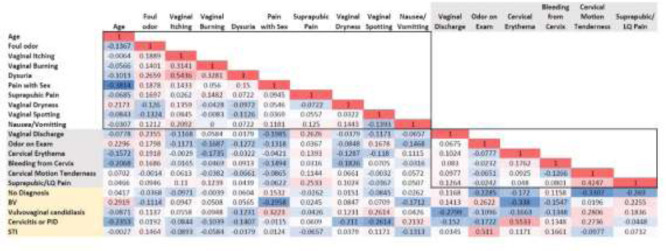
Spearman Correlations of Symptoms, Physical Exam Findings, and Diagnoses.

Vaginal itching, burning, dysuria, and pain with sex were positively correlated, with vaginal itching, burning and dysuria having strong correlations (0.30 to 0.54) (Figure 1). Symptoms were weakly correlated with each other except for cervical motion tenderness and suprapubic/lower quadrant pain (0.43). Most physical exam findings were also weakly correlated with each other (<0.20). There were weak correlations between reported symptoms and physical exam findings, though foul discharge was positively correlated with vaginal discharge on exam (0.24), cervical erythema (0.21). Reported suprapubic pain correlated with suprapubic/lower quadrant pain on exam (0.23), vaginal spotting with odor on exam (0.23), vaginal burning inversely correlated with odor on exam (-0.22).

Red represents positive correlation and blue represents negative correlation with intensity of color representing strength of correlation. Symptoms are listed first, followed by physical exam findings (shaded grey), and diagnoses (shaded yellow). Boxes are placed around symptoms and around exam findings, to facilitate within and between group comparisons. Diagnoses are not clustered with each other as most women had single diagnosis.

### Variability in Physician Diagnosis by History and Physical Exam Findings

**Figure 2:**
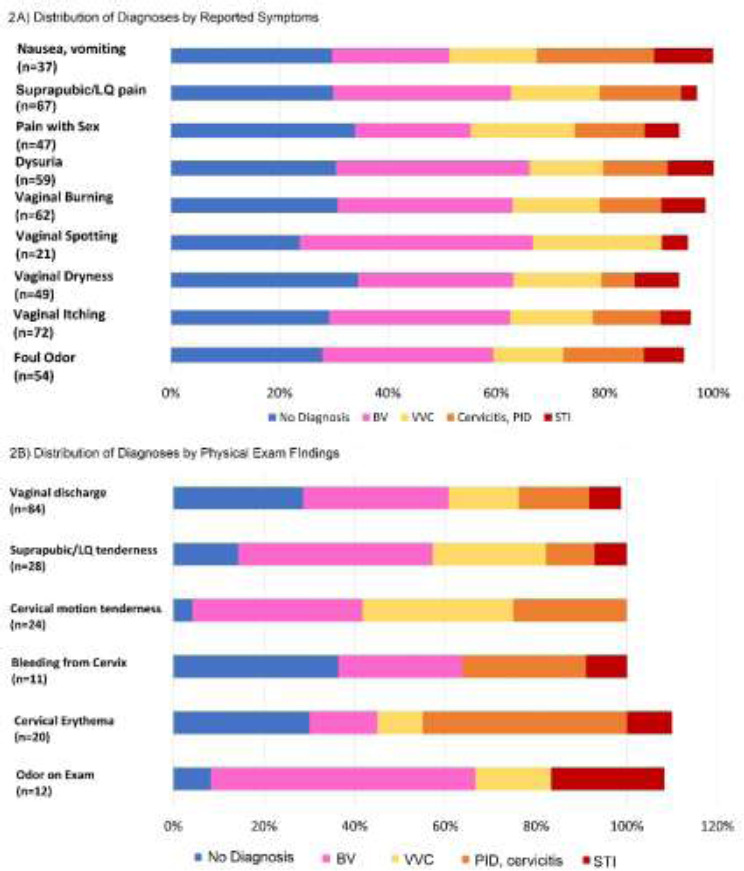
Distribution of Diagnoses by (A) Reported Symptoms and (B) Physical Exam Findings.

Physician diagnosis was documented for 91 (93%) participants. Of those with available diagnosis, 31% were given a diagnosis of normal physiologic vaginal discharge (n=26) or no diagnosis (n=3 “vaginal discharge unspecified”, n=1 “N/A”). The next most common diagnosis was BV (31%), followed by vulvovaginal candidiasis (15%), cervicitis/PID (13%), and STI (7%). The distribution of provider diagnosis by symptoms and exam findings are shown in Figure 2. Several findings from history point to a non-physiologic diagnosis, including report of foul odor, vaginal itch, dysuria, and dyspareunia, but no reported symptoms strongly differentiated diagnostic categories.

Diagnoses varied considerably by exam findings with cervical motion tenderness, odor on exam, and suprapubic/lower quadrant tenderness more frequently having a specific diagnosis, and women presenting with vaginal discharge, friability or cervical erythema having greater frequency of no specific diagnosis. Unsurprisingly, exam findings most strongly positively correlated with cervicitis/PID were cervical erythema (0.56) and cervical motion tenders (0.28). Odor on exam was most strongly correlated with diagnosis of STI (0.47) and BV (0.30). Vaginal discharge on exam did not have strong correlation with any diagnosis, and this may be due to the ubiquity of the finding

The distribution of diagnoses by (A) reported symptoms and (B) physical exam findings are shown above, with frequency on the x-axis and symptom or exam finding on the y-axis.

## Discussion

Our study illustrates the diagnostic dilemma of vaginal discharge syndrome, which is not readily available in the current body of literature for Haiti. We observed substantial variability in diagnosis by history and physical examination findings, and limited correlation between history and physical exam findings.

Etiologic testing has superior sensitivity and specificity compared to syndromic management for vaginal discharge. However, when etiologic testing is not possible, syndromic management can improve diagnostic accuracy of vaginal and cervical infections, when combined with speculum examination. In meta-analysis of worldwide studies, pooled diagnostic sensitivity was assessed for four flowcharts: (1) history and risk assessment, (2) history, risk assessment, and speculum examination, (3) prior elements plus Gram stain and microscopy of vaginal discharge samples, (4) country adapted flowcharts or flowcharts not fitting prior descriptions. The pooled diagnostic sensitivity for cervical infections ranged from 27.4% - 90.1%, and 53.9-93.3% for vaginal infections with BV or *Trichomonas vaginalis*, and sensitivity was increased with addition of speculum examination and microscopy (Zemouri, 2016).

Results from our study support the utility of speculum examination, as it correlated more strongly with diagnoses, but also the need for standardization as the weak correlations could represent variability in assessment and documentation of findings. In addition to standardization, we note that physical exam findings as documented, could be more comprehensive, such as including assessment and documentation of genital warts, inguinal lymphadenopathy, adnexal tenderness, cervical ectopy, and discharge from os. Standardization of exams and documentation may be supported through inclusion of these pre-determined elements in charts, and in-services for clinicians to come to consensus on how they are assessing and documenting the exam findings. Lastly, development of local algorithms for when speculum exam is available/feasible and when it is not, can promote standardization of care and treatment according to syndromic management guidelines.

We did not conduct etiologic testing for gonorrhea, chlamydia, trichomoniasis, or BV as we were unable to find a local laboratory capable of running the tests. Of note, in the study by Bristow et al. conducted in rural Haiti in 2013, specimens for chlamydia and gonorrhea testing via nucleic acid amplification test (NAAT) were transported after 5 days to Port-au-Prince, where they underwent testing within 2 weeks, and then women who had not been treated empirically were followed up. It is unlikely that this would be sustainable or scalable, especially under conditions of increased instability in Haiti, thus highlighting the need for improved syndromic management of vaginal and cervical infections. Systems limitations including the absence of a lab in the clinic facility, no regional labs with NAAT capability and no public health infrastructure for notification of lab results and contact tracing result in insurmountable barriers to reliance on lab testing for diagnosis and treatment.

Important limitations of our study must be acknowledged. Our sample size was modest and a convenience sample of when the research assistant was available to obtain informed consent. As such, we do not overextend the data with attempts to model or control for factors such as age, symptom duration, or sexual risk, and present results descriptively. Despite these limitations, this data is essential for representing the limitations of sexual and reproductive health care in this extremely resource constricted setting. Future studies to better characterize infectious etiologies of vaginal discharge syndrome with rapid tests that don’t require laboratories would be ideal. Currently, we recommend standardized and expanded symptom and physical exam reporting, differentiation strategies, and empiric treatment recommendations.

Currently in survival mode, the Jerusalem Clinic is struggling to keep its doors open. The primary care physician in charge and co-author, Dr. Fiquita St. Paul, is unable to travel to clinic on many days due to safety concerns. The route to clinic is ruled by gangs; kidnapping, rape and murder on the route are routine. When she can make it to clinic, she contends with supply chain disruptions and limited lab diagnostics as described above. Equipping her and other providers with standardization of clinical exam findings and documentation as well as development of treatment algorithms according to syndromic management guidelines will improve accuracy and antibiotic stewardship. This can be accomplished with virtual in-services, electronic medical record (EMR) build-out and chart reviews with feedback. In addition, having standardization around clinical exam findings, documentation and adherence to syndromic management will improve the quality and consistency of care provided.

### Declaration of Conflicting Interests

The authors declare that there is no conflict of interest associated with this study.

**List of Abbreviations:**
STI: (Sexually transmitted infection)
CT: (*Chlamydia trachomatis*)
NG: (*Neisseria gonorrhoeae*)
TV: (*Trichomonas vaginalis*)
BV: (Bacterial vaginosis)
LMIC: (low- and middle- income countries)
PID: (Pelvic inflammatory disease)

## References

[1] Bristow CC, Desgrottes T, Cutler L (2014). The aetiology of vaginal symptoms in rural Haiti. International Journal of STD & AIDS.

[2] Community Empowerment Global Health Partnerships:Jerusalem, Haiti.

[3] (2021). Guidelines for the management of symptomatic sexually transmitted infections.

[4] Peebles K, Velloza J, Balkus JE (2019). High Global Burden and Costs of Bacterial Vaginosis:A Systematic Review and Meta-Analysis. Sexually Transmitted Diseases.

[5] Unemo M, Ross J, Serwin AB (2020). European guideline for the diagnosis and treatment of gonorrhoea in adults [published online ahead of print, 2020 Oct 29]. International Journal of STD & AIDS.

[6] UNICEF (2023). Country Profiles.

[7] van Gemert C, Hellard M, Bradshaw CS (2018). Syndromic management of sexually transmissible infections in resource-poor settings:a systematic review with meta-analysis of the abnormal vaginal discharge flowchart for Neisseria gonorrhoea and Chlamydia trachomatis. Sexual Health.

[8] Web Annex 1. Key data at a glance (2021). In:Global progress report on HIV, viral hepatitis and sexually transmitted infections. Accountability for the global health sector strategies 2016-2021:actions for impact.

[9] Workowski KA, Bachmann LH, Chan PA ((2021)). Sexually Transmitted Infections Treatment Guidelines. Center for Surveillance, Epidemiology, and Laboratory Services, Centers for Disease Control and Prevention (CDC), U.S. Department of Health and Human Services, Atlanta, GA.

[10] Zemouri C, Wi TE, Kiarie J (2016). The Performance of the Vaginal Discharge Syndromic Management in Treating Vaginal and Cervical Infection:A Systematic Review and Meta-Analysis. PLOS One.

